# Current Trends and Future Directions in the Diagnosis and Management of Pediatric Orthopedic Disorders

**DOI:** 10.7759/cureus.102904

**Published:** 2026-02-03

**Authors:** Mohammed H Al-Rumaih, Abdulrahman F Al-Otaibi, Tareq S Almukhlafi

**Affiliations:** 1 Department of Orthopaedic Surgery, Security Forces Hospital Program, Riyadh, SAU

**Keywords:** 3d-printing, artificial intelligence, clubfoot, developmental dysplasia of the hip, management, pediatric fractures

## Abstract

Congenital disorders, such as clubfoot, developmental dysplasia of the hip (DDH), and developmental problems, such as scoliosis, traumatic fractures, infections, tumors, neuromuscular dysfunction, and sports injuries, are all pediatric orthopedic disorders that impact millions of children around the world and can cause a lifetime of musculoskeletal disability unless they are treated early. These disorders are caused by genetic, biomechanical, and environmental factors that affect developing bones and joints. The review is a synthesis of recent developments during the 2018-2025 period, drawing on major studies from major databases.

Radiation-free methods, such as high-resolution ultrasound in DDH, low-dose cone-beam computed tomography (CBCT) in fractures, fast magnetic resonance imaging (MRI) protocols, and artificial intelligence (AI)-based models have been shown to improve diagnosis with 90-98% accuracy in fracture detection, scoliosis classification, and bone age assessment. Trends in management focus on minimally invasive, growth-preservation strategies. The Ponseti technique and Pavlik harnesses remain very effective for clubfoot and DDH, and surgeries for scoliosis are minimized with magnetically controlled growing rods. Bioabsorbable fixation, virtual surgical planning (minimizing operating time and fluoroscopy), and biologics like platelet-rich plasma help promote improved healing with fewer complications.

The innovations will reduce morbidity and improve long-term outcomes by providing personalized, evidence-based care. Nevertheless, the issues persist, including the lack of AI validation, access disparities in low-resource environments, and the need for more rigorous multicenter trials. The future outlook is validated AI integration, regenerative stem cell therapies, 3D-printed personalized implants, robotics, and genetic treatment to bring care to all children more equitably and effectively.

## Introduction and background

Pediatric orthopedic conditions occur in millions of children and adolescents annually and cause serious complications of musculoskeletal health [1]. Such disorders extend to the congenital anomalies, e.g., clubfoot, developmental dysplasia of the hip (DDH), developmental disorders, e.g., scoliosis and slipped capital femoral epiphysis (SCFE), traumatic injuries (e.g., fractures), infectious processes (osteomyelitis, septic arthritis), neoplastic disorders (e.g., osteosarcoma, Ewing sarcoma), neuromuscular disorders (e.g., cerebral palsy), and sport-related trauma (anterior cruciate ligament (ACL) tears and osteochondritis dissecans). The causes of these disorders include genetic, biomechanical, and environmental factors that influence growth plates (physes), resulting in long-term deformities unless treated in time [2]. DDH, as an example, causes instability of the femoral head and acetabular malformation [3]. Likewise, scoliosis is established as a developmental disorder characterized by a three-dimensional spinal curvature that may progress during growth spurts [4]. Traumatic fractures, comprising approximately 30% of the injuries that children sustain, frequently involve the upper extremity and should be considered in terms of physeal involvement to prevent growth distractions [1]. As per the epidemiological statistics, congenital anomalies and injuries alone account for 14.6% of the low- and middle-income countries’ pediatric surgical cases [5]. DDH is approximated at 1-20 per 1000 live births, with a higher rate observed in females [2].

Diagnostic procedures have been optimized to enhance accuracy and safety within the field. Radiography is a necessity, supplemented by radiation-sparing modalities to safeguard growing tissues [1]. High-resolution ultrasound has become a staple for early detection of DDH, with a sensitivity of over 90% [1,2]. Moreover, magnetic resonance imaging (MRI), including perfusion MRI, is associated with disease severity and prognosis in Legg-Calve-Perthes disease [6], and low-dose cone-beam computed tomography (CBCT) reduces radiation exposure by 78% during fracture evaluation [1]. Artificial intelligence (AI) based on deep learning algorithms, like convolutional neural networks, achieves more than 90% accuracy in scoliosis, fracture (AUC 0.844-0.876), and bone age (98.6% in 1 year) [7]. Next-generation sequencing (NGS) and polymerase chain reaction (PCR)-based culture-negative infection panels are molecular diagnostics with a sensitivity of more than 90% and are useful in polymicrobial cases [8]. In addition, 3D printing technology has enhanced diagnosis by visualizing complex abnormalities, such as a hemi-vertebrae in congenital scoliosis, leading to the planning of patient-specific care to minimize surgical risks and operative time [9].

Non-operative surgery includes the use of the Pavlik harness in DDH and dynamic bracing in scoliosis, where patients have a choice and can avoid surgery, when possible [1,10]. Among surgical innovations are magnetically guided growing rods (MCGRs) used to correct scoliosis and robot-aided screw implantation to improve accuracy [11]. Biologics used in the treatment of osteochondritis dissecans include platelet-rich plasma and mesenchymal stem cells, and guided growth plates aim to reduce deformities over time [12]. Short-course antibiotic therapy, in which intravenous treatment is replaced by oral therapy with less invasive drainage, helps patients recover from infections such as osteomyelitis and septic arthritis more quickly [13]. Endoprostheses 3D-printed and vascularized fibula transfers enhance the likelihood of limb preservation in children with bone tumors [9]. AI can help in management by predicting risks such as venous thromboembolism and surgical outcomes [7].

Recent improvements, e.g., AI and diagnostic accuracy, are essential to reduce morbidity and improve skeletal integrity through more individualized interventions that consider unique developmental patterns to minimize complications [1,3,7]. However, significant research gaps remain, including a lack of familiarity with AI among orthopedic surgeons working with children, limited external model validation, data heterogeneity, and ethical concerns (privacy, equal access). AI and other technologies are not yet available in LMICs, and the primary diagnostic aid is still traditional radiography. The purpose of this review is to present a general overview of the current trends, recent findings in the main pediatric orthopedic disorders, and the future of diagnosis and treatment of these disorders.

## Review

Methodology

This review was conducted in a structured way to integrate modern developments in the assessment and treatment of pediatric orthopedic conditions. Four major databases, Web of Science, PubMed, and Scopus, were searched for publications published from 2018 to 2025. This period was selected for its emphasis on contemporary trends, such as the blistering adoption of AI and regenerative therapies, which have been on the rise over the past several years.

 The main search terms were combinations of “pediatric orthopedic disorders,” “developmental dysplasia of the hip,” “scoliosis,” “pediatric fractures,” “congenital limb deformities,” “osteomyelitis,” “cerebral palsy,” “regenerative medicine,” “artificial intelligence,” “minimally invasive surgery,” and “3D printing.” The keywords were selected thoughtfully and connected with Boolean operators (e.g., AND, OR) to broaden the scope while focusing on the details of the topic.

Titles and abstracts were screened to remove irrelevant studies, and full-text screening was conducted to assess relevance and quality. Preference was given to peer-reviewed papers such as randomized controlled trials, multicenter cohorts, systematic reviews, and high-quality observational studies. Animal research, case reports (not including new methods), research in adults, and non-English literature were excluded.

Studies considering children below the age of 18 years, research prioritizing diagnosis, management, or progress of pediatric orthopedic conditions, original research or superior reviews, and the use of human subjects were considered inclusion criteria. After screening, 32 articles met these criteria and formed the basis of this review, providing a balanced representation of evidence across key topics.

Results and discussion

Developmental Dysplasia of the Hip (DDH)

DDH refers to a spectrum of hip joint abnormalities that include mild acetabular shallowness, up to acetabular dislocation [14]. It depends on the population and location. It occurs with an incidence ranging between 1 and 20 per 1,000 live births [2], based on its risk factors, including breech presentation (4-8 fold increased risk of incidence), female sex, family history, and oligohydramnios [15].

A standardized sonographic method known as the Graf method is the gold standard for diagnosing infants under six months of age. Universal ultrasound screening programs, such as those in Austria and Germany, have largely reduced late-presenting cases to fewer than 0.5 per 1,000 live births, driven by high levels of early intervention [1]. The research by Kraus et al. on the long-term program in Austria emphasized its efficiency in reducing the number of surgical interventions and hospitalizations through regular screening using the Graf method [16]. Physical tests, such as the Ortolani and Barlow maneuvers in newborns, and the replacement of X-rays after six months, are now also being incorporated into modern diagnostics [17].

Management centers on conservative, age-appropriate methods in young cases. In infants with reducible hips below 6 months of age, the Pavlik harness remains first-line, achieving 85-95% success in recent cohorts [1,18]. Dynamic braces, such as the Tübingen hip flexion splint, have similar or better success rates and lower rates of avascular necrosis. A systematic review comparing established tubing with better outcomes on milder Graf grades and shorter treatment in certain series [19]. Continued follow-up is necessary because early prevention can significantly reduce the risk of osteoarthritis.

Scoliosis and Spinal Deformities

Scoliosis is a three-dimensional spinal disorder characterized by lateral curvature, rotation, and frequent sagittal disproportion in children, with etiologies including idiopathic, congenital, neuromuscular, and syndromic [20]. The most common form is adolescent idiopathic scoliosis (AIS), with genetic factors (such as polymorphisms in GPR126 and the calmodulin pathway), female sex (2-3 times higher risk), familial history, and environmental factors (poor posture and low BMI) as the most common multifactorial causes. Congenital malformations or neuromuscular diseases are frequently associated with early-onset scoliosis (EOS, younger than 10 years), which presents more risks to pulmonary and thoracic development [20,21].

Recent advances in diagnosis favor radiation-free procedures, such as 3D surface topography (3DST) monitoring, to assess progression without repeat X-rays. Wang et al. demonstrated the 3DST's sensitivity of 87.8% for identifying a curve change of 5 degrees or greater in children [22]. This method is becoming increasingly popular in clinics as part of regular follow-up.

The management focuses on bracing, which enables active children to tolerate sports to enhance compliance. Kuang et al. reported that contemporary braces incorporate designs that are flexible in daily use, thereby lowering dropout rates [23]. Noninvasive outpatient lengthenings using magnetically controlled growing rods (MCGRs) permit external magnetic lengthenings, which dramatically reduce the need for repeated surgeries compared with traditional growing rods (TGRs) [24]. In a comparative study of 5-year follow-up in idiopathic EOS, MCGRs had better curve correction (38 vs. 46 major curve) and reduced complications and revisions (risk ratio 2.1-2.2 lower than TGRs), but no difference in spinal growth or quality-of-life scores [11]. A different Food and Drug Administration (FDA) investigational device exemption (IDE) study with a 3-10 year follow-up (mean 6.6 years) of 57 participants demonstrated long-term thoracic Cobb correction from 40.4 to 22.1, with 71% at 30 years and above, yet 18% may require revision [25].

Clubfoot and Limb Deformities

Clubfoot or congenital talipes equinovarus (CTEV) is a frequent congenital musculoskeletal disorder in which the foot is inverted and downwardly rotated, which can limit mobility without treatment [26]. It has a worldwide prevalence of approximately 1.3% of live births, and multifactorial origins include genetic (alterations in genes such as TBX4, PITX1, and HOX clusters that govern limb formation), intrauterine mechanical restrictions, and possible environmental factors [27].

Advanced imaging has enhanced early detection in diagnosis. Prenatal ultrasound, notably 3D/4D ultrasound, allows the observation of foot position and minor defects, and authors report detection rates of over 85% in high-volume centers at 18-22 weeks of gestation [28]. After birth, treatment is guided by clinical examination using the Pirani scoring system, and ultrasound and MRI provide detailed images of soft tissues and cartilage without radiation [28]. Artificial intelligence in LLD measurement is on the rise, where radiographs are automated to reduce errors in children's clinics. Lezak et al. have noted the use of AI to standardize LLD detection as telemedicine use increases [29].

The Ponseti method, a minimally invasive approach that includes serial casting, percutaneous Achilles tenotomy, and bracing, is at the heart of management. In 91 patients (146 feet) treated between 2011 and 2023, initial correction was observed in all (100%), with excellent long-term outcomes in 77.7% (mean follow-up 54 months), but relapsed in 22.2%, necessitating further interventions [30].

In congenital limb deformities other than clubfoot, such as fibular hemimelia or angular deformities, guided growth methods, such as tension-band plates, are used to correct defects at 0.5-1° per month in skeletally immature patients [31]. In complex cases, distraction osteogenesis, typically using motorized intramedullary nails, such as PRECICE, is used to treat length discrepancies. Masci et al. established the safety of PRECICE in pediatric lengthening, allowing controlled correction at 1 mm/day with a lower risk of infection than with external fixators [31].

Slipped Capital Femoral Epiphysis (SCFE)

SCFE is a common pediatric hip condition with posterior and inferior displacement of the epiphysis of the head of the femur in relation to the metaphysis via the growth plate. The incidence of the condition is estimated at 10.8 per 100,000 children [32]. Among these risk factors are obesity (including BMI above the 95th percentile), male sex, and endocrine disorders like hypothyroidism [32]. The diagnosis is increasingly based on advanced imaging to detect it at an early stage. MRI is relevant in identifying pre-slip and early physeal pathology, such as widening, marrow edema, and peri-physeal signal abnormalities that are not apparent on plain radiographs [32].

Surgical stabilization is used by management to avoid additional slippage, where new innovations focus on accuracy and fewer complications. In stable slips, in situ pinning using a single cannulated screw is standard and may be fully threaded to enhance purchase. Pediatric applications of bioabsorbable screws have been considered, providing initial stability equivalent to metal and avoiding future hardware removal [33]. Severe or unstable slips frequently demand more vigorous correction. The modified Dunn technique allows anatomic realignment by surgically dislocating it. In a meta-analysis, a total AVN rate of 10.3% was reported using the modified Dunn method, with the highest rates in unstable cases but a tendency toward lower odds in stable slips relative to in situ pinning [34].

Pediatric Fractures

Pediatric fractures are fractures of bones in children, and their cause is often attributed to high-energy trauma, such as falls in play, sports injuries, or accidents. The unique characteristics of immature bones that are more elastic and porous explain their appearance as greenstick or buckle fractures instead of fractures viewed in adults [35]. A diagnostic tool is low-dose cone-beam computed tomography (CBCT), which reduces radiation levels considerably; however, its image quality cannot be compared with that of other technologies for measuring complex fractures [36]. Iterative reconstruction algorithms also produce higher-quality images at lower doses, enabling safer serial imaging of complex injuries. These characteristics render CBCT especially useful for intra-articular fractures, such as distal humerus fractures, or for transitional ankle injuries [37].

Moving to management, contemporary trends favor single-limb spica casts for treating femoral fractures in children to enhance mobility and ease caregiving without compromising stability. Even bioabsorbable fixation is being pursued to avoid secondary surgeries to remove hardware, especially in forearm and ankle injuries. The most recent developments in management include virtual surgical planning (VSP), in which Trisolino et al. observed that it led to a 13-shot reduction in fluoroscopy and a 45-minute decrease in operative time in pediatric cases with lower limb deformities, using patient-specific instruments [38]. In a study by Hamdy et al., comparing single-limb and one-and-a-half spica casts in 84 children, the authors reported no significant difference in bone union time or complications, but higher parental satisfaction with the single-limb device due to better hygiene and comfort in the children (P < 0.001) [39].

In terms of outcomes, meta-analyses of operative management of both-bone forearm fractures show an overall refracture rate of 5.5 percent, with no distinguishing results between intramedullary nailing and plate fixation [40].

Infectious Conditions (Osteomyelitis and Septic Arthritis)

Pediatric osteomyelitis and septic arthritis are severe bone and joint infections in children, most caused by bacteria in the bloodstream (acute hematogenous route), with common offenders, including *Staphylococcus aureus* (including MRSA strains) and, in younger children, *Kingella kingae*. These include trauma, recent infections anywhere in the body, or immune weakness that provides bacteria the opportunity to seed the growing skeleton [1,41]. Recent developments in diagnostics aim to quickly and precisely identify pathogens to inform treatment. Multiplex PCR panels, such as the BioFire Joint Infection panel, have 93% sensitivity in culture-negative osteoarthritis and identify 33 targets, including bacteria and resistance genes, in less than an hour. An analysis of BioFire in 50 pediatric patients demonstrated that it could detect culture-negative cases, shortening time to specific antibiotics and hospitalization [42]. MRI is also essential for early diagnosis, and Dobaria and Cohen (2023) highlight its high contrast resolution for detecting bone marrow edema and abscesses within three to five days of symptom onset, compared to radiographs [43].

The management has changed to shorter antibiotic courses and timely intervention to conserve functioning. This 2023 Infectious Diseases Society of America (IDSA) guideline recommends three to seven days of initial IV antibiotics, followed by oral transition, in uncomplicated cases, supported by RCTs showing similar efficacy with prolonged IV treatment [44]. Wu et al. reported a low recurrence rate of 3.6% at a median of 2 years when courses were shortened, attributing success to early debridement and antimicrobials [45]. It is recommended that mobilization between 48 and 72 hours after the drain is necessary to avoid stiffness. Autogenous bone grafting is useful in refractory cases to restore defects.

Future directions and recommendations

The future of pediatric orthopedics remains promising, with AI tools in multicenter trials to aid in access equity in other populations. Saini et al. highlighted that substantial research is needed to confirm AI's diagnostic and predictive accuracy in practice, minimize bias, and improve outcomes for underserved children [1]. The field of regenerative medicine is advancing rapidly, particularly with stem cell therapies used to repair growth plate damage and deformity. Aloman et al. highlighted the need for effective animal models that reduce bony bar formation and, subsequently, for human experiments that could restore normal growth [46].

The 3D printing of individual implants will become more popular, allowing customization of their use in case of complex deformities and minimizing complications when expanding the bone. Benady et al., in their article on 3D printing in pediatric orthopedics, noted the expansion of 3D printing to preoperative planning and patient-specific guides and recommended further advancement of bioresorbable materials [9]. Smart sensors and robotics will improve monitoring and precise surgery.

Workforce shortages are also a pressing issue, and, according to forecasts, gaps persist in rural regions despite any training. An analysis of the workforce suggested expanding telemedicine and specialized training programs to reduce disparities [47]. In the case of congenital deformities, genetic therapies, such as vosoritide, are still in development and have demonstrated that more precise positioning and fewer corrective surgeries are required in achondroplasia when used in combination with early intervention [48]. In general, these guidelines emphasize cross-disciplinary collaboration, high-quality evidence, and equal implementation to change the care of children with musculoskeletal problems.

## Conclusions

Pediatric orthopedics has made significant strides, and AI diagnostics, rapid molecular testing, minimally invasive surgery, and child-friendly implants have improved outcomes for children worldwide. Since early screening for DDH and scoliosis, shorter antibiotic courses for infections, and bioabsorbable solutions for fractures, these innovations detect problems earlier, minimize complications, and improve growth and function. The Ponseti technique, Pavlik harness, and magnetically controlled rods are considered child-centered and achieve long-term outcomes. In the future, validating new technologies, making them accessible globally, and incorporating regenerative and genetic therapies will build a better future, providing every child with an opportunity to lead a healthier, more active life.

## References

[1] Saini J, Kaushik N, Jain P, Kumar J, Singh B (2025). Recent advances in the diagnosis and management of pediatric orthopedic disorders: a comprehensive review. Cureus.

[2] Singh S (2025). Congenital and developmental orthopedic disorders in children: advances in detection, management, and future directions. J Contemp Clin Pract.

[3] Ghandour M, Klotz M, Horsch A (2023). Orthopedics and trauma in children: key problems and future insights. Children (Basel).

[4] Sung S, Chae HW, Lee HS (2021). Incidence and surgery rate of idiopathic scoliosis: a nationwide database study. Int J Environ Res Public Health.

[5] Espinoza P, Mwakyembe T, Kajoka HD (2025). Epidemiology, outcomes, and access to care for pediatric patients who underwent surgery in Northern Tanzania: a cross-sectional study. PLOS Glob Public Health.

[6] Kim HK, Wiesman KD, Kulkarni V (2014). Perfusion MRI in early stage of Legg-Calvé-Perthes disease to predict lateral pillar involvement. A preliminary study. J Bone Joint Surg Am.

[7] Zech JR, Ezuma CO, Patel S (2024). Artificial intelligence improves resident detection of pediatric and young adult upper extremity fractures. Skeletal Radiol.

[8] Gupton M, Burns J (2023). Metagenomic next-generation sequencing in osteoarticular infections with a focus on pediatrics: current concepts and clinical applications. EFORT Open Rev.

[9] Benady A, Gortzak Y, Ovadia D (2025). Advancements and applications of 3D printing in pediatric orthopedics: a comprehensive review. J Child Orthop.

[10] İşçi H, Özdemir Görgü S (2025). Understanding bracing outcomes in adolescents with idiopathic scoliosis: a mixed-methods approach. Front Rehabil Sci.

[11] Saarinen AJ, Flynn JM, Thompson GH, Emans JB, Sturm PF, Sponseller PD, Helenius IJ (2026). Traditional versus magnetically controlled growing rods for idiopathic early-onset scoliosis: outcomes at 5-year follow-up. Spine J.

[12] Kuznetsov AS, Kozhevnikov OV, Kralina SE (2023). Correction of proximal femoral deformities in children by a guided growth technique: a review. Pediatr Traumatol Orthop Reconstr Surg.

[13] Dutta S, Kabra NS, Saini SS (2025). Shorter or biomarker-guided antibiotic durations for common serious neonatal infections: a collection of non-inferiority meta-analyses. EClinicalMedicine.

[14] Vaquero-Picado A, González-Morán G, Garay EG, Moraleda L (2019). Developmental dysplasia of the hip: update of management. EFORT Open Rev.

[15] Lankinen V, Helminen M, Bakti K, Välipakka J, Laivuori H, Hyvärinen A (2023). Known risk factors of the developmental dysplasia of the hip predicting more severe clinical presentation and failure of Pavlik harness treatment. BMC Pediatr.

[16] Kraus T, Chiari C (2024). Universal screening for developmental dysplasia of the hip in Austria: what have we learned?. Explor Musculoskeletal Dis.

[17] Cosgrove AP, Maizen C (2025). Detection and treatment of developmental dysplasia of the hip in infants: updates and recommendations. Curr Opin Pediatr.

[18] Bakarman K, Alsiddiky AM, Zamzam M, Alzain KO, Alhuzaimi FS, Rafiq Z (2023). Developmental dysplasia of the hip (DDH): etiology, diagnosis, and management. Cureus.

[19] Nair A, Yatsonsky D, Liu J (2024). Comparison of outcomes of different Graf grades of developmental dysplasia of the hip in infants treated with Tubingen splint versus Pavlik harness - a systematic review. J Orthop.

[20] Johnson AN, Lark RK (2024). Current concepts in the treatment of early onset scoliosis. J Clin Med.

[21] Wang S, Li M, Ren J, Tao J, Fang M, Kong L (2025). Global prevalence and associated risk factors of scoliosis in children and adolescents: a systematic review and meta-analysis. BMC Public Health.

[22] Wang H, Zhu Y, Bao Q, Lu Y, Yan F, Du L, Qin L (2025). A novel portable and radiation-free method for assessing scoliosis: an accurate and reproducible study. BMC Musculoskelet Disord.

[23] Kuang H, Chen L, Huang M, Chen J (2025). Management of adolescent scoliosis: a comprehensive review of etiology and rehabilitation. Front Pediatr.

[24] Jamnik AA, Shaw KA, Thornberg D, McClung A, Jo CH, Ramo B, McIntosh A (2024). Health-related quality of life and clinical outcomes for magnetically controlled growing rod patients after treatment termination. Spine Deform.

[25] Oh T, Patel M, Nice E, Pahys JM, Hwang SW, Samdani AF (2025). Results of a retrospective FDA investigational device exemption study for anterior vertebral body tethering in adolescent idiopathic scoliosis with 3-year to 10-year follow-up. JB JS Open Access.

[26] Smythe T, Owen RM, Aspden A (2025). Global clubfoot treatment in 2023: an overview of advances and outcomes. BMJ Glob Health.

[27] Umar M, Tong L, Jin H, Terebessy T, Chen D (2025). Genetics, epidemiology and management of clubfoot and related disorders. Genes Dis.

[28] do Amaral E Castro A, Peixoto JB, Miyahara LK (2024). Clubfoot: congenital talipes equinovarus. Radiographics.

[29] Lezak BA, Pruneski JA, Oeding JF (2024). Diagnostic performance of deep learning for leg length measurements on radiographs in leg length discrepancy: a systematic review. J Exp Orthop.

[30] Rastogi A, Agarwal A (2021). Long-term outcomes of the Ponseti method for treatment of clubfoot: a systematic review. Int Orthop.

[31] Masci G, Palmacci O, Vitiello R (2021). Limb lengthening with PRECICE magnetic nail in pediatric patients: a systematic review. World J Orthop.

[32] Johns K, Mabrouk A, Tavarez M (2025). Slipped capital femoral epiphysis. StatPearls [Internet].

[33] Thaiparambil S, Ahuja A, Kalsoom U, Bagadia B, Nair A (2025). Comparison of management strategies and associated complications with slipped capital femoral epiphysis. Cureus.

[34] Abdelnasser MK, Hassan AA, Ibrahim M, Ibrahim AH, Abol Oyoun N (2025). Does the development of AVN after the modified Dunn procedure for slipped capital femoral epiphysis (SCFE) depend on slip stability? A systematic review and meta-analysis. BMC Musculoskelet Disord.

[35] van Bergen CJ, Colaris JW (2024). Shedding light on pediatric fractures: bridging the knowledge gap. Children (Basel).

[36] Demehri S, Baffour FI, Klein JG (2023). Musculoskeletal CT imaging: state-of-the-art advancements and future directions. Radiology.

[37] Kraus M, Pertman L, Eshed I (2025). What's new in pediatric musculoskeletal imaging. J Child Orthop.

[38] Trisolino G, Depaoli A, Menozzi GC (2023). Virtual surgical planning and patient-specific instruments for correcting lower limb deformities in pediatric patients: preliminary results from the in-office 3D printing point of care. J Pers Med.

[39] Hamdy MS, Sabry AO, Helmy BA, Hana AZ, El Zawahry AM, Gamal A (2025). Comparative study between single-limb versus one-and-a-half hip spica cast in fracture femur in young children. J Pediatr Orthop.

[40] Syed AN, Ashebo L, Lawrence JT (2024). Refracture following operative treatment of pediatric both bone forearm fractures. J Pediatr Orthop.

[41] He M, Arthur Vithran DT, Pan L, Zeng H, Yang G, Lu B, Zhang F (2023). An update on recent progress of the epidemiology, etiology, diagnosis, and treatment of acute septic arthritis: a review. Front Cell Infect Microbiol.

[42] Udaondo C, Alcobendas Rueda RM, Diaz-Delgado B, Remesal A, Quiles-Melero I, Calvo C (2024). Clinical utility of a multiplex PCR panel (BioFire Joint Infection®) in the adjustment of empiric antimicrobial therapy: experience in pediatric osteoarticular infections. Children (Basel).

[43] Dobaria DG, Cohen HL (2023). Osteomyelitis imaging. StatPearls [Internet].

[44] Woods CR, Bradley JS, Chatterjee A (2024). Clinical practice guideline by the Pediatric Infectious Diseases Society (PIDS) and the Infectious Diseases Society of America (IDSA): 2023 guideline on diagnosis and management of acute bacterial arthritis in Pediatrics. J Pediatric Infect Dis Soc.

[45] Wu H, Wang S, Shen J (2025). Clinical features and sequelae of pediatric osteomyelitis following surgical treatment: a 10-year retrospective study. BMC Infect Dis.

[46] Aloman C, Stone D, Sankar R, Thabet-Hagag A (2025). Mesenchymal stem cells in pediatric physeal growth arrest: a systematic review. Cureus.

[47] Cho RH, Sheffer BW, Shah AS (2025). Pediatric orthopaedic workforce in 2022: current workforce, projections, and trends in the United States. J Pediatr Soc North Am.

[48] Allegri AE, Bedeschi MF, Bocchi MB (2025). Integrating vosoritide therapy with limb surgery in paediatric patients with achondroplasia: real-life experiences. Orphanet J Rare Dis.

